# PEG10 as an oncogene: expression regulatory mechanisms and role in tumor progression

**DOI:** 10.1186/s12935-018-0610-3

**Published:** 2018-08-13

**Authors:** Tian Xie, Shan Pan, Hang Zheng, Zilv Luo, Kingsley M. Tembo, Muhammad Jamal, Zhongyang Yu, Yao Yu, Jing Xia, Qian Yin, Meng Wang, Wen Yuan, Qiuping Zhang, Jie Xiong

**Affiliations:** 1 Department of Immunology, School of Basic Medical Science, Wuhan University, Wuhan, 430071 China; 2 Department of Urology, Zhongnan Hospital of Wuhan University, Wuhan, 430071 China; 3 Michael Chilufya Sata Hospital, Mpika, Zambia; 4 State Key Laboratory of Agriculture Microbiology, Huazhong Agricultural University, Wuhan, 430070 China; 5 Hubei Provincial Key Laboratory of Developmentally Originated Disease, Wuhan University, Wuhan, 430071 China

**Keywords:** PEG10, Regulatory mechanisms, Proliferation, Apoptosis, Metastasis, Targeted therapies

## Abstract

Cancer is a major public health problem as one of the leading causes of death worldwide. Deciphering the molecular regulation mechanisms of tumor progression can make way for tumor diagnosis and therapy. *Paternally expressed gene 10* (*PEG10*), located on human chromosome 7q21.3, has turned out to be an oncogene implicated in the proliferation, apoptosis and metastasis of tumors. PEG10 has been found to be positively expressed in a variety of cancers with seemingly complex expression regulation mechanisms. In this review, we focus on the most vital factors influencing PEG10 expression and recapitulate some of the currently known and potential mechanisms of PEG10 affecting tumor progression, as understanding the molecular regulatory mechanisms of tumor progression can provide potential PEG10 related diagnosis and biomarker specific targeted therapies.

## Background

*Paternally expressed gene 10* (*PEG10*), shows 61.4% homology with murine *myelin expression factor 3* (*MyEF*-*3*), which encodes a distinctive protein functioning as a transcriptional factor during brain development. Conservation of CCHC-type zinc finger motif suggests that PEG10 may also function as a transcriptional factor [1–3]. *PEG10* gene is highly conserved in eutherian mammals which indicates its essential functions, and its protein shows high similarity to retroviral gag-pol proteins [4, 5]. Researches on mice suggest that *peg10* plays an important role in placenta formation and adipocyte differentiation, and its knockout can cause embryonic lethality [5, 6]. PEG10 is strongly expressed in placenta, ovary and testis as well as somatic adult organs including brain, kidney and lung [1, 7–9]. However, dysregulated expression of PEG10 has been closely associated with the cell proliferation, apoptosis and development of malignancies. However, the comprehensive mechanism of the regulation of PEG10 expression is still in its infancy.

This review will be an update on the current state of PEG10 in cancer related researches. We try to gain insights into the factors mostly influencing PEG10 expression and the profound mechanisms of PEG10 in tumor progression and potential PEG10 related therapeutic targets.

## The structure of *PEG10* gene

*PEG10* is derived from the Ty3/Gypsy retrotransposon family which is located on human chromosome 7q21.3 in a head-to-head orientation with another paternally expressed gene *SGCE*. There is an 800 bp CpG (cytosine-phosphate-guanine) island between *SGCE* and *PEG10*. Li et al. [10] found epigenetic silencing of *PEG10* by promoter methylation led to the low expression of PEG10 mRNA, but the activation of *PEG10* was not necessarily associated with hypomethylation. Suzuki et al. [11] analyzed the CpG island methylation status and found that the methylation started about 60 bp downstream from the transcription start site of PEG10, suggesting that it is the methylation of downstream regulatory elements rather the promoter methylation that inhibits maternal transcription.

**Fig. 1:**
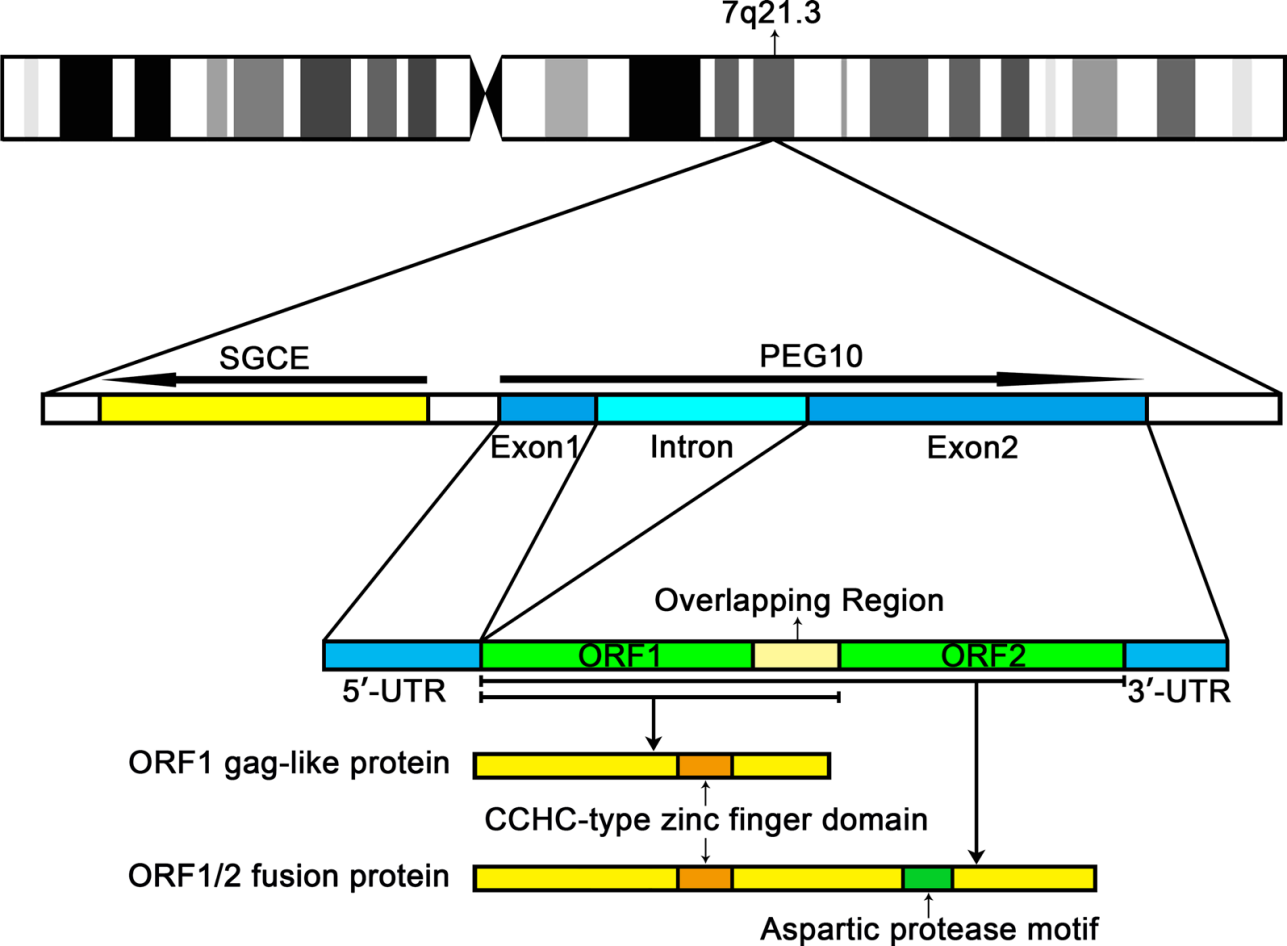
**Genomic information of human PEG10 gene.** PEG10 gene is located on the q21.3 of chromosome 7. The bold arrows show the orientations of PEG10 and SGCE. Exon 1 transcribes the 5’-UTR and exon 2 transcribes two ORFs and the 3’-UTR. ORF1 encodes a gag-like PEG10-RF1 protein, which contains a CCHC-type zinc finger domain. This component protein regulates cell proliferation and apoptosis as a main functional protein. By -1 frameshift translation, the ORF1 and the ORF2 translated PEG10-RF1/2 fusion protein with an aspartic protease motif

*PEG10* gene consists of two exons, separated by a 6.8 kb intron, and its major transcript is 6639 bp (NM_001172438.2) [12]. The exon 1 of *PEG10* contains the 5′-untranslated region (UTR) and exon 2 contains two overlapping open reading frames (ORFs) and a 4 kb 3′-UTR sequence [13]. The ORF1 codes for the gag-like PEG10-RF1 protein with a coiled-coil domain in N-terminal and a zinc finger domain in C-terminal, while the pol-like PEG10-RF2 protein is synthesized by a programmed-1 frameshift translation. During the programmed − 1 frameshifting, the ORF1 and the ORF2 translated a gag-pol-like fusion protein named PEG10-RF1/2 from one mRNA (Fig. 1) [4, 14]. Evidence suggests that the translation of PEG10 is also initiated at CUG codon except for the conventional AUG codon [12]. This new finding will add novel cognition to the PEG10’s − 1 frameshifting translation mechanism.

## The expression levels of PEG10 in cancers

### The expression levels of PEG10 in normal and cancer tissues

**Fig. 2:**
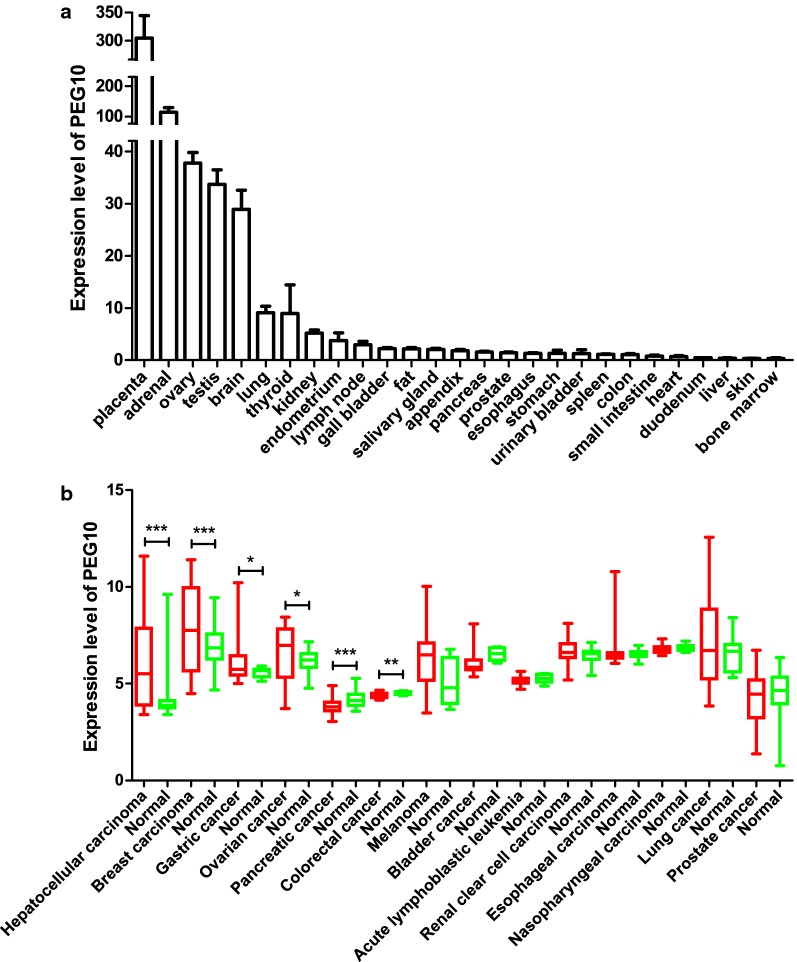
**Differential expression pattern of PEG10 in normal tissues and cancers.** **a**) The expression levels of PEG10 in normal tissues. PEG10 is relatively highly expressed in placenta, adrenal, ovary, testis and brain, but shows pretty low expression levels in other normal tissues.** b**) The expression levels of PEG10 in cancers. PEG10 is overexpressed in HCC and breast cancer, but downregulated in pancreatic carcinoma and colorectal cancer. *P < 0.05, **P < 0.01, ***P < 0.001 by Student’s *t* test

We searched NCBI Gene database to access the expression levels of PEG10 in normal tissues [15]. As shown in Fig. 2a, PEG10 is relatively highly expressed in placenta, adrenal, ovary, testis and brain, but the expression levels are pretty low in other tissues, which are consistent with the previous literature [1, 7–9]. As shown in Table 1, several studies have reported PEG10 is positively expressed in a variety of cancers such as hepatocellular carcinoma (HCC) [9, 16–19], pancreatic carcinoma [20], breast cancer [10], prostate cancer [10], gallbladder carcinoma [21], thyroid cancer [22], oral squamous cell carcinoma [23], colon cancer [24], enchondromas [25] and B-cell chronic lymphocytic leukemia (B-CLL) [26]. Worth noting was that the amplification of *PEG10* gene copy numbers detected in HCC also contributed to PEG10 overexpression [17, 27–29].

**Table 1:** The expression of PEG10 in cancer patients and cancer cell lines

Tumors	Type	Methods	Expression	References
HCC	Primary HCC	IHC	15/16^a^	[9]
	HCC tissues and cell lines	cDNA microarray	Significantly higher	[16]
	HCC cell lines	QPCR	18/20^b^	[17]
	Primary HCC	QPCR	Significantly higher	
	HCC tissues	IHC	148/218^a^	[19]
Pancreatic carcinoma	Pancreatic carcinoma tissues	IHC	85/160^b^	[20]
Breast cancer	Ductal carcinoma	IHC	6/11^a^	[10]
	Invasive ductal carcinomas	IHC	7/22^a^	
	Breast cancer tissue (n = 161)	IHC	36%^a^	
Prostate cancer	Prostate cancer tissue (n = 30)	IHC	37%^a^	[10]
Gallbladder carcinoma	Gallbladder adenocarcinoma tissues	IHC	52/108^a^	[21]
Thyroid cancer	24 thyroid cancer tissues and 14 normal thyroid tissues	QPCR	Significantly higher (P = 0.034)	[22]
OSCC	OSCC tissues	QPCR	83/118^b^	[23]
Colon cancer	Colon cancer tissues	QPCR and WB	9/20^a^	[24]
Enchondromas	Enchondromas tissues	IHC and QPCR	Strongly positive expression	[25]
B-CLL	B-CLL PBMNCs	QPCR	40/42^b^	[26]

*IHC* immunohistochemistry, *QPCR* quantitative polymerase chain reaction, *OSCC* oral squamous cell carcinoma, *WB* western blot, *PBMNC* peripheral blood mononuclear cell

^a^Proportion of PEG10-positive tumor tissues/cells

^b^Proportion of tumor tissues/cells occurred PEG10 upregulation compared to normal tissues/cells

To further confirm the expression levels of PEG10 in cancers, we used Gene Expression Omnibus (GEO) datasets to analyze [30]. As shown in Table 2 and Fig. 2b, we observed that PEG10 was overexpressed in several cancers especially HCC and breast cancer. However, PEG10 was shown to be downregulated in pancreatic carcinoma and colorectal cancer, which may be contradictory with the previous literatures. These contradictory results may be due to the data we analyzed were at mRNA level, which may not represent the protein level exactly. In addition, although PEG10 was not shown to be overexpressed in some tumors with regard to the result we analyzed, it may be highly expressed in some specific tumor subtypes since the results we showed are overall. For example, Akamatsu et al. [31] reported that PEG10 was upregulated in neuroendocrine tumors such as neuroendocrine prostate cancer and lung cancer but no significant upregulation in other subtypes.

**Table 2:** Basic information of the 14 GEO datasets

Cancer type	Accession number	Number of samples (tumor/normal)	P-value	References
*Upregulated*				
Hepatocellular carcinoma	GSE14520	233/233	< 0.0001	[57, 58]
Breast carcinoma	GSE10780	42/143	0.0002	[59]
Gastric cancer	GSE13861	71/19	0.0388	[60]
Ovarian cancer	GSE14407	12/12	0.0438	[61]
*Downregulated*				
Pancreatic cancer	GSE28735	45/45	0.0003	[62, 63]
Colorectal cancer	GSE32323	17/17	0.0011	[64]
*Insignificant*				
Melanoma	GSE3189	45/7	0.0848	[65]
Bladder cancer	GSE3167	41/9	0.1243	[66]
Acute lymphoblastic leukemia	GSE26713	117/7	0.2168	[67]
Renal clear cell carcinoma	GSE36895	23/23	0.2445	[68]
Esophageal carcinoma	GSE23400	53/53	0.2526	[69, 70]
Nasopharyngeal carcinoma	GSE12452	31/10	0.2710	[71–73]
Lung cancer	GSE30219	293/14	0.2938	[74]
Prostate cancer	GSE6919	58/58	0.7478	[75, 76]

P-values were acquired through t-test for the comparison of PEG10 expression between cancer and normal

### PEG10 overexpression is correlated with poor clinicopathological characteristics

Many studies have shown that the expression of PEG10 is closely related to the prognosis of clinicopathological characteristics. Ge et al. [32] conducted meta-analysis to systematically evaluate the correlations between PEG10 and the clinicopathological characteristics in patients with solid tumors. They found that PEG10 overexpression was associated with the higher risk of solid tumors incidence, lower degree of differentiation, increased lymph node metastasis and advanced TNM stage. Moreover, a high level of PEG10 expression was closely correlated to poor overall survival (OS) and it could be used as an independent prognostic biomarker for patients with solid tumors. Furthermore, Bang et al. [19] indicated that PEG10 protein could be a potential biomarker for predicting early recurrence and recurrence-free survival (RFS) in HCC patients after curative resection, even in those with normal serum α-fetoprotein levels.

### Factors regulating the expression levels of PEG10

The expression level of PEG10 is regulated by many factors. Transcription factors like E2F, c-MYC and androgen receptor (AR) have been reported to participate in PEG10 expression regulation. Wang et al. [18] reported that both E2F-1 and -4 could directly bind to the PEG10 promoter, and upregulate its transcription in HCC, which was confirmed by chromatin immunoprecipitation (ChIP) and dual luciferase report assay. E2F-1’s direct upregulation of PEG10 expression via binding to the *PEG10* promoter was also verified in prostate and pancreatic cancer [18, 20, 31]. Besides, in lung cancer cells, GSK3β/USP11/E2F-1/PEG10 pathway was shown to play an imperative role in PEG10 overexpression [33]. Li et al. [10] reported that c-MYC knockdown in Panc1 cells resulted in a subsequent PEG10 downregulation, and ChIP assays validated that *PEG10* was a direct downstream target of c-MYC. In prostate cancer, AR was confirmed to bind to the *PEG10* promoter region, thus repressing the transcription of PEG10. Treating prostate cancer cells with synthetic androgen R1881 resulted in an increased AR occupancy at the *PEG10* promoter, while decreased when treated with AR antagonist Enzalutamide [31].

Transforming growth factor β (TGF-β) signaling pathway plays a biphasic role in cancer progression [34]. In HCC, PEG10 was increased after treating HepG2 cells with TGF-β1 [44]. However, the mutual inhibition effect of PEG10 and TGF-β signaling was found in chondrosarcoma and enchondroma [25]. Shinohara et al. proposed that TGF-β might inhibit PEG10 expression through the downregulation of c-MYC [10, 25, 35]. Besides, Wang et al. suggested that TGF-β might also inhibit the expression of PEG10 by keeping Rb dephosphorylated to inhibit the release of E2F [18, 36].

Several miRNAs have also been proven to regulate the expression level of PEG10. In HCC, miR-122 repressed the translation level of PEG10 via directly binding to sites 2310 and 2403 in PEG10 3′-UTR [37, 38]. Additionally, miR-491 has also been confirmed to negatively regulate the expression of PEG10 directly in colorectal cancer [24].

## Functions of PEG10 and the mechanisms thereof

### PEG10 promotes tumor proliferation

Uncontrolled proliferation is an important factor in tumor progression. Numerous researches have reported that PEG10 plays a significant role in promoting the cancer proliferation. The proliferation ability of cancer cells was improved after overexpressing PEG10 in HCC, while decreasing endogenous expression of PEG10 showed prominent growth retardation [9]. PEG10’s role in proliferation was also confirmed in colorectal cancer cell line HCT-116, as Curcumin was able to diminish the proliferation effect by upregulating the expression of miR-491 [24]. In addition, our data revealed that the proliferation ability of Raji cells and A549 cells was decreased after being transfected with PEG10 siRNA [39, 40], and that PEG10 promoted breast cancer cell proliferation after being overexpressed [41]. The function of PEG10 was also certified in gastric cancer, where knockdown of PEG10 in MKN7 cells reduced anchorage-independent colony formation [42]. Additionally, it has also been reported that GSK3β increases the interaction of E2F1 with USP11, which results in the deubiquitination and stabilization of E2F1, which in turn activates PEG10 expression to promote proliferation in A549 [33]. Furthermore, c-MYC protein binds to E-box sequences in the first *PEG10* intron and activates its transcription, which further promotes the proliferation of several tumor cells [10, 33]. In vivo experiments showed that volume and weight of the tumors obtained from xenograft tumorigenicity assays were both lower and the Ki-67 score reduced significantly after PEG10 knockdown [20, 31]. The underlying mechanism of PEG10 promoting the proliferation may be due to its cell-cycling promoting effect. In pancreatic and neuroendocrine prostate cancers, the levels of p21, p27 (cell-cycle-dependent kinase inhibitors) and Cyclin E1 (which accumulates at G0/G1 to S phase and reduced smoothly from S to G2/M phase) were upregulated after PEG10 knockdown, which indicate PEG10 drives cell cycle progression from G0/G1 to S phase [20, 31].

### PEG10 inhibits the apoptosis of cancer cells

Apoptosis is a physiological process of programmed cell death, which is indispensable in cell development and homeostasis [43]. Dysfunction in apoptosis pathways is a typical characteristic of cancer cells. As an oncogene, *PEG10* also has been shown to play an anti-apoptosis role in cancer progression. In Raji cells and HCT-116 cells, PEG10 was found to inhibit the apoptosis of cancer cells [24, 39], while in HepG2 cells PEG10 was shown to increase Bcl-2 expression and decrease Bax expression, and diminish apoptosis induced by doxorubicin [44]. PEG10 overexpression decreases cell death mediated by SIAH1 in HCC, while SIAH1 also reduces the amount of PEG10 protein, thus inducing growth arrest and apoptosis in hepatoma cells [9, 45]. In addition, mRNA interference of PEG10 in human hepatocyte L02 cells resulted in elevated expression levels of anti-apoptosis protein BCL-xL [18]. Similarly, our early studies have also demonstrated that in B-cell acute lymphoblastic leukemia (B-ALL) and B-cell chronic lymphocytic leukemia (B-CLL), CXCL13 and CCL19 together upregulate PEG10 expression in CD23^+^CD5^+^ or CD19^+^CD34^+^ B cells and then hamper the activation of caspase-3 and caspase-8 to gain the apoptosis resistance induced by tumor necrosis factor-α (TNF-α) [46, 47].

### PEG10 promotes metastasis of cancer cells

**Fig. 3:**
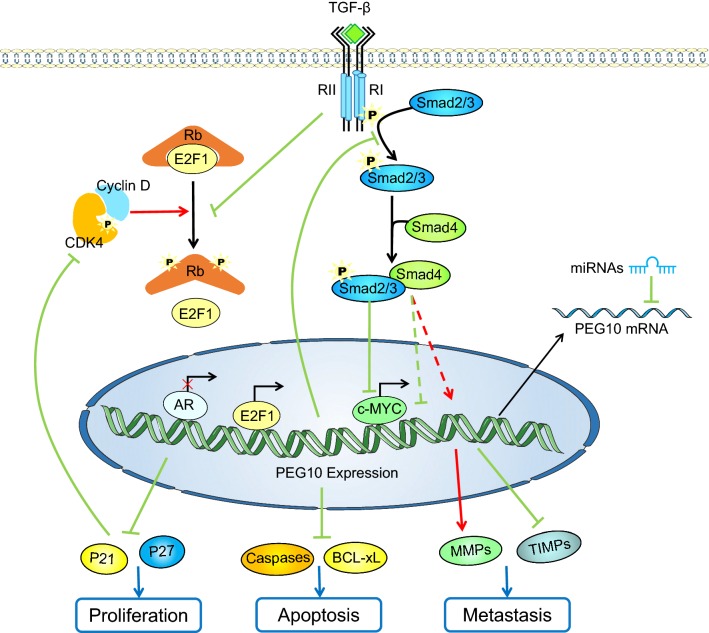
**Schematic representation of PEG10 expression regulation mechanisms and its role in tumor progression.** miRNAs indicate miR-122 and miR-491; Caspases indicate caspase 3 and caspase 8; MMPs indicate MMP-1, -2 and -9; TIMPs indicate TIMP-1 and -2. The solid arrows indicate that these signaling pathways have been confirmed; the dashed arrows show that these signaling pathways need to be further validated

Tumor metastasis is an important factor in promoting poor prognosis in cancer patients and is mainly characterized by the migration, invasion and epithelial–mesenchymal transition (EMT) of cancer cells. It has been shown that PEG10 is overexpressed in metastatic prostate cancer and rectal adenocarcinoma versus primary and benign tumors, thus indicating that PEG10 may be involved in cancer metastasis [31, 48]. Our studies revealed that suppressing the expression of PEG10 in human Raji cells and A549 cells resulted in the reduction of migration and invasion capabilities of cells, and the decrease of matrix metalloproteinases (MMPs) like MMP-2 and -9 [39, 40]. In addition, we also observed that overexpression of PEG10 promoted the migration and invasion of breast cancer cell line MDA-MB-231 cells, in which the expression levels of MMP-1, -2 and -9 were increased while that of TIMP-1 and -2 decreased [41]. Besides, PEG10 also turned out to promote pancreatic cancer cells migration and invasion through ERK/MMP7 pathway [20]. In HCC, overexpression of PEG10 in HepG2 cells decreased the expression levels of epithelial marker protein E-cadherin and increased the expression levels of mesenchymal marker protein vimentin. PEG10 may be involved in the activation of canonical TGF-β pathway to promote the EMT of cancers. Although PEG10 could inhibit TGF-β receptors to block TGF-β pathway and may diminish the inhibition of proliferation induced by TGF-β signaling, the invasion and EMT induced by TGF-β could be removed after PEG10 knockdown [44]. In prostate cancer cells, after TGF-β treatment, PEG10 knockdown decreased Smad2 and Smad3 phosphorylation, SBE-4 (which contains four copies of Smad binding elements) luciferase reporter activity and decreased the expression of mesenchymal transcription factor Snail1 and Zeb1 (which are the direct mediators of the TGF-β pathway) [31]. A series of clinical investigations revealed that HCC [19], gallbladder cancer [21, 49], lung cancer [40], oral squamous cell carcinoma [23], pancreatic cancer [20] and gastric cancer [42] are more vulnerable to metastasis or invasion with PEG10 overexpression. A diagram of the expression regulation factors of PEG10 and the underlying mechanisms of the oncogenic role of PEG10 in cancer progression is shown in Fig. 3.

## Therapy

### Immunotherapy of HCC

Recent years have witnessed the rapid development of carcinoma immunotherapy. Dendritic cells (DCs) are powerful professional antigen presenting cells which were discovered in 1973 and have been used in anti-tumor immunotherapy since the 1980s. In 2010, the first therapeutic tumor DCs vaccine sipuleucel-T for the treatment of metastatic prostate cancer was approved by the US Food and Drug Administration (FDA) based on its confirmed safe and non-toxic side effects [50]. After being transfected with recombinant adenovirus (Ad) vectors encoding tumor associated antigen (TAA), DCs are able to process the TAA to peptides and then bind them to MHC class I molecules for recognition by CD8^+^ T cells. Besides, DCs express costimulators to provide the signals needed for differentiation of CD8^+^ T cells into anti-tumor specific cytotoxic T lymphocytes (CTLs) which are able to recognize and kill tumor cells without a requirement for costimulation [50–53].

Numerous evidence demonstrated that PEG10 was overexpressed in HCC and contributed to the oncogenesis, thus it might be a TAA of HCC. Peng et al. [54] transfected DCs with PEG10 recombinant Ad and found the DCs could specifically elicit CTLs to secrete interferon-γ (IFN-γ) and lyse HepG2. This Ad-PEG10 transduced DCs could induce an anti-tumor immune response against PEG10 positive HCC with HLA-A2 restricted both in vitro and in vivo. The findings indicate that DCs transfected with Ad-PEG10 might be an ideal target for HCC immunotherapy.

### Others

In recent years, there have been growing interest in RNA-targeted therapies [55]. As described above, PEG10 may be a target for intervention in cancer. Using antisense oligonucleotides or siRNA to interfering PEG10 mRNA for treatment can be taken into consideration. Besides, to further explore the mechanisms of PEG10 promoting tumor progression and find it is which domain of PEG10 protein that actually works, may pave the path for the design of the small molecular inhibitors which may also be an effective strategy for PEG10 inactivation. In addition, Kempinska et al. [56] demonstrated that the menin–MLL1 complex binds to PEG10 gene directly and catalyzes H3K4me3 to upregulate the expression of PEG10 through the epigenetic mechanism. The menin–MLL inhibitor MI-503 showed an anti-tumor effect of HCC, and the underlying mechanism may be owing to the indirect downregulation of PEG10. Therefore, exploring the agents that can inhibit the function of PEG10 indirectly may also be a potential approach for tumor therapy.

## Conclusions

*PEG10* is an imprinting gene that plays a key role in tumor proliferation, apoptosis and metastasis. The studies of PEG10 in tumors mainly focused on HCC, but in recent years extensive studies have shown that PEG10 also contributes to the progression of many types of cancer. The expression level of PEG10 is regulated by several factors, but their effects may vary in different tumors. Since PEG10 is highly expressed in tumors, it may serve as a TAA which can be utilized in tumor immunotherapy or a potential target for new anti-cancer regimens and cancer diagnosis. Nevertheless, further studies are needed to gain insights into the molecular mechanisms of the role PEG10 played in cancer.

## Abbreviations

PEG10: paternally expressed gene 10
MyEF-3: murine myelin expression factor 3
CpG: cytosine-phosphate-guanine
UTR: untranslated region
ORF: open reading frame
HCC: hepatocellular carcinoma
B-CLL: B-cell chronic lymphocytic leukemia
GEO: Gene Expression Omnibus
OS: overall survival
RFS: recurrence-free survival
AR: androgen receptor
ChIP: chromatin immunoprecipitation
GSK3β: glycogen synthase kinase-3β
USP11: ubiquitin-specific protease 11
TGF-β: transforming growth factor β
B-ALL: B-cell acute lymphoblastic leukemia
TNF-α: tumor necrosis factor-α
EMT: epithelial–mesenchymal transition
MMPs: matrix metalloproteinases
TIMPs: tissue inhibitor of metalloproteinases
DCs: dendritic cells
Ad: adenovirus
TAA: tumor associated antigen
CTLs: cytotoxic T lymphocytes
IFN-γ: interferon-γ
MLL1: mixed lineage leukemia 1
H3K4me3: trimethylation of H3K4
